# Prediction of a key role of motifs binding E2F and NR2F in down-regulation of numerous genes during the development of the mouse hippocampus

**DOI:** 10.1186/1471-2105-7-367

**Published:** 2006-08-02

**Authors:** Michal Dabrowski, Stein Aerts, Bozena Kaminska

**Affiliations:** 1 Laboratory of Transcription Regulation, Department of Cell Biology, The Nencki Institute of Experimental Biology, Pasteura 3, 02-093 Warsaw, Poland; 2 Laboratory of Neurogenetics, Department of Human Genetics, VIB and Katholieke Universiteit Leuven, Herestraat 49, 3000 Leuven, Belgium

## Abstract

**Background:**

We previously demonstrated that gene expression profiles during neuronal differentiation *in vitro *and hippocampal development *in vivo *were very similar, due to a conservation of the important second singular value decomposition (SVD) mode (Mode 2) of expression. The conservation of Mode 2 suggests that it reflects a regulatory mechanism conserved between the two systems. In either dataset, the expression vectors of all the genes form two large clusters that differ in the sign of the contribution of Mode 2, which for the majority of them reflects the difference between down- or up-regulation.

**Results:**

In the current work, we used a novel approach of analyzing *cis*-regulation of gene expression in a subspace of a single SVD mode of temporal expression profiles. In the putative upstream regulatory sequences identified by mouse-human homology for all the genes represented in either dataset, we searched for simple features (motifs and pairs of motifs) associated with either sign of the loading of Mode 2. Using a cross-system training-test set approach, we identified E2F binding sites as predictors of down-regulation of gene expression during hippocampal development. NR2F binding sites, for the transcription factors Nr2f/COUP and Hnf4, and also NR2F_SP1 pairs of binding sites, were predictors of down-regulation of expression both during hippocampal development and neuronal differentiation. Analysis of another dataset, from gene profiling of myoblast differentiation *in vitro*, shows that the conservation of Mode 2 extends to the differentiation of mesenchymal cells. This permitted the identification of two more pairs of motifs, one of which included the CDE/CHR tandem element, as features associated with down-regulation both in the differentiating myoblasts and in the developing hippocampus. Of the features we identified, the E2F and CDE/CHR motifs may be associated with the cycling progenitor cell status, while NR2F may be related to the entry into differentiation along the neuronal pathway.

**Conclusion:**

Our results constitute the first prediction of an expression pattern from the genomic sequence for the developing mammalian brain, and demonstrate a potential for the analysis of gene regulation in a subspace of a single SVD mode of expression.

## Background

Transcriptome profiling experiments documented changes in gene expresion in several areas or cellular populations of the developing mammalian brain [1-4]. The availability of genomic sequences of an increasing number of vertebrate species, including human and mouse, permits – by their comparison – the identification of putative regulatory sequences on the scale of whole genomes [5,6]. Analysis of regulatory sequences together with data from expression profiling holds promise of computational identification of features of regulatory sequences associated with particular patterns of expression [7,8].

The motifs present in corresponding *cis*-regulatory sequences of orthologous genes are often the same, but their precise arrangement may be changed [9,10]. Such conserved motifs are more likely to correspond to functional motifs, namely transcription factor binding sites [11]. Pairs of closely situated conserved motifs are particularly attractive candidate features, as pairs of closely situated transcription factor binding sites were postulated to constitute minimal units or "composite elements" contributing to specific patterns of gene regulation [12,13].

Regulatory regions of genes of metazoans, including mammals, have hierarchical and subunit organization [14]. Promoters and enhancers are built of *cis*-regulatory modules (CRM), which in turn are built of transcription factor binding sites. Importantly for the computational approach used here, some of these subunits have largely independent and approximately additive (in a log scale) effects on expression. Examples of such subunits include: different enhancers of the same gene [15], certain CRMs of well studied promoters [14,16], multiple instances of the same motif, or even different motifs, within the same promoter [7,17]. Effects of independent and additive mechanisms on regulation of many genes can be conveniently analyzed after transformation of the expression data from the original basis of time-points to an orthogonal basis. In this new basis, the temporal expression profiles are regarded as weighted sums of orthogonal (uncorrelated) modes, analogous to the modes of a vibrating violin string. Decomposition of temporal expression profiles into orthogonal modes, by singular value decomposition (SVD) [18] was described before [19,20].

Hippocampal neuronal culture [21] is a well-established experimental system, in which a wealth of information on almost every aspect of neuronal differentiation has been obtained. In particular, this system is relevant for studies of gene regulation during the neuronal differentiation, because the expression profiles *in vivo *are highly similar to those *in vitro *[22]. Apparently, once the cells have taken a neuronal fate, the remainder of the gene expression program is relatively autonomous. Another advantage of this system for studies of gene regulation is a relative ease of experimental manipulations [23,24], which opens a prospect of verification of the predictions generated *in silico *by experimental work.

In a recent paper [25], we applied the SVD analysis to the expression data from neuronal differentiation *in vitro *[22] and hippocampal development *in vivo *[2]. We demonstrated that the high correlation observed between the *in vitro *and *in vivo *expression profiles stems from the conservation of a single SVD mode (Mode 2) of temporal expression profiles. In both systems, Mode 2 was the most important among the modes that carried information about the relative changes of expression in time. The contributions (loadings) of Mode 2 were highly conserved across the 453 genes that were common to both datasets, suggesting that this mode reflects an underlying regulatory mechanism conserved between the two systems. A loading of Mode 2 to the expression of a particular gene corresponded to a component of a continous decrease (for the negative loading), or a continuous increase (for the positive loading) to the expression vector of this gene. In both compared datasets, the expression vectors of the genes form two large clusters – differening by the sign of the contribution of Mode 2, which, for the majority of the genes, reflects the difference between down- or up-regulation in the course of neuronal differentiation and hippocampal development.

In the current work, we used a novel approach of analyzing *cis*-regulation of gene expression in a subspace of a single conserved SVD mode of temporal expression profiles. Instead of looking for common patterns of expression in the whole space of expression measurements, we considered a common pattern of expression in an orthogonal subspace of the original measurement space with just a single SVD mode as its basis vector. For this analysis we choose the previously characterized Mode 2. In the putative upstream regulatory sequences identified by mouse-human homology, we sought to identify simple features (motifs and pairs of motifs) associated with either sign of the loading of Mode 2 (up- or down-regulation). Conservation of Mode 2 permitted the use of a cross-system training-test set approach, where only the most promising features that were selected in one dataset were tested on the other dataset. This way we identified E2F binding sites as features predictive of down-regulation of gene expression during hippocampal development, and Nr2f/COUP binding sites, and NR2F_SP1 pairs of binding sites as features predictive of down-regulation during both hippocampal development and neuronal differentiation. Addition of another datset to the comparison – from the gene profiling of myoblast differentiation *in vitro*, demonstrated that the conservation of Mode 2 extends to the differentiation of cells of mesenchymal origin, and led to the identification of two features associated with down-regulation of gene expression during hippocampal development and myoblast differentiation.

## Results

Both the neuronal and the hippocampal dataset can be split (partitioned) into two roughly equal sets of genes by the loading sign of Mode 2 (Table 1). These two sets correspond to the two clusters of expression vectors in the subspace of the first two modes previously reported [25]. Here, we sought to identify features of the putative regulatory sequences associated with gene membership in either of the two clusters. We assumed that the conservation of Mode 2 demonstrated previously for the genes common between the two datasets extends to all the genes in either dataset. In our feature search, we used the expression data and the putative regulatory sequence data of all the genes in either dataset (i.e. not only the common genes) for which we identified at least one putative regulatory sequence (approximately half of the genes in either dataset) – see Table 1.

We employed a candidate feature approach, with 148 non-redundant motifs conserved between mouse and human (hereafter referred to as motifs), and their 10878 possible pairs (co-occurrences), in putative upstream regulatory regions, used as the candidate features. Identification of the conserved non-coding sequences (CNSs) used as the putative regulatory sequences, and of conserved non-redundant motifs present in those sequences were performed using established tools, as described in Methods. For each candidate feature, we evaluated its association with the sign of Mode 2, by calculating the probability of the split of the set of CNSs harboring this feature between the sets of CNSs assigned to either sign of the loading of Mode 2. The split ratio (odds) expected for each feature under the *H*_0 _hypothesis of no association is equal to the split ratio in the general population of all CNSs, i.e. approximately 1:1 (Table 1). The more asymmetric the split observed for a particular feature and the larger the group of CNSs containing this feature, the less likely is this split under the *H*_0 _hypothesis, thus permitting the identification of the most promising features.

We took advantage of the conservation of Mode 2 between the two datasets [25], and employed a training-test set approach, where one dataset is used as training set and the other as the test set. During training all possible features of a given type were ranked according to the significance of their association with the sign of Mode 2, and 10 highest-ranking features (with the smallest p-values on the training set) were selected (feature selection) for testing on the other dataset (cross-system test). During the testing, the same statistics (p-value) as during the training was used, but this time calculated on the other (test) dataset. The training and test were performed for both possible choices of the training and testing dataset (neuronal for training and hippocampal for testing, and the other way round).

To address the issue of multiple testing we used the Bonferroni correction. The p-values obtained on the training set were multiplied by the number of features of a given type scored during the training (148 for motifs, 10878 for all their possible pairs). The p-values obtained on the test set for the 10 features earlier selected on the training set were multiplied by 10.

### Single motifs

**Figure 1:**
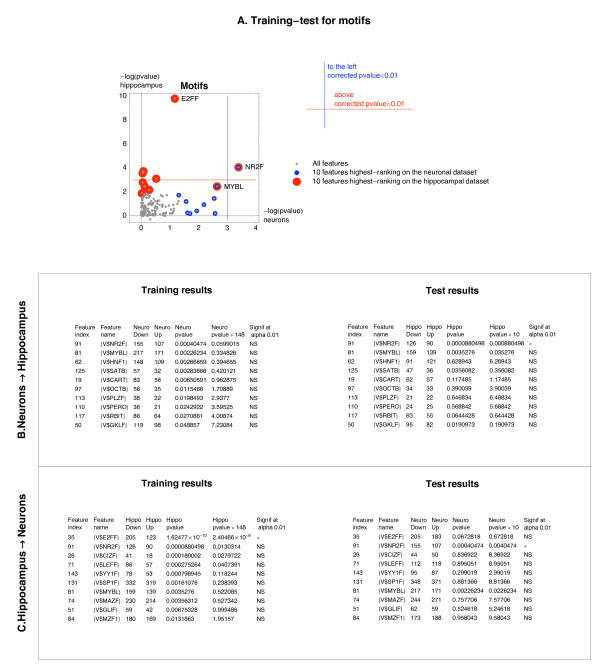
**Identification of motifs associated with the sign of Mode 2 during hippocampal development and/or neuronal differentiation**. The ten most promising motifs associated with the sign of Mode 2 selected on the neuronal dataset were tested on the hippocampal dataset. The same procedure was repeated for the reverse choice of the training and the test dataset (hippocampal dataset used for training, neuronal for testing). **A**. Illustration of the training-test procedure. The X and Y coordinates represent single-test p-values computed for each feature on the neuronal and on the hippocampal dataset, shown as -log_10_(p-value). The 10 highest-ranking features on the neuronal dataset are indicated by blue dots, and the 10 highest-ranking features on the hippocampal dataset are indicated by red dots. The vertical (blue) line, and the horizontal (red) line mark the alpha thresholds of the corrected p-value 0.01 in the cross-system test, on the neuronal and the hippocampal dataset, respectively. The feature is significant in the cross-system test if a blue dot is above the red line, or a red dot is to the left of the blue line. The dots representing the significant features are labeled with motif names. A double coloring of a dot (blue on red) indicates a feature that was among the 10 highest ranking features on both the training and the test dataset – such features are also labeled. A dummy p-value of 2 was used to mark features absent in either dataset. **B**. Results of the training on the neuronal dataset and the test on the hippocampal dataset. "Feature name" corresponds to the Genomatix "matrix family name". "Down" and "Up" indicate the numbers of CNSs assigned to the genes with the negative and the positive sign of Mode 2. **C**. Results of the training on the hippocampal dataset and the test on the neuronal dataset.

When the neuronal dataset was used for training and the hippocampal dataset for testing, of the 10 highest-ranking motifs selected on the neuronal dataset, all associated with down-regulation, the NR2F motif scored as significantly associated with down-regulation (corrected p-value < 0.01) during the test on the hippocampal dataset (Figure 1A, B).

Notably, the same NR2F motif was identified as significantly associated with down-regulation for the reverse choice of the training and test dataset (Figure 1A, C). The presence of the NR2F motif in a CNS was associated with odds 1.44:1 (155:107) and 1.4:1 (126:90), on the neuronal and hippocampal dataset, that this CNS would be assigned to a gene with a negative loading of Mode 2. These odds correspond to odds ratios 1.55:1 and 1.72:1, respectively, when compared with the odds in the general population of all the CNSs in a given dataset (Table 1). The NR2F motif corresponds to a Genomatix-defined family [26] of 6 matrices describing binding sites for the transcription factor families Hnf4 (Hnf4, Hnf4g) and Nr2f/COUP (Nr2e3, Nr2f1, Nr2f2), known to bind to overlapping sets of sites. Two motifs, namely NR2F and MYBL were common between the sets of motifs selected during the training on the neuronal and the hippocampal dataset (Figure 1A). The MYBL motif would be significant for both directions of the cross-system test at alpha level 0.05. This motif corresponds to a family of 7 matrices describing binding sites for the transcription factors from the Myb family (Myb, Mybl1, Mybl2).

During the training on the hippocampal dataset we identified the E2FF motif as being most significantly associated with down-regulation (corrected training p-value of 2.4 × 10^-8^, Figure 1C) The presence of the E2FF motif in a CNS was associated with odds of 1.67:1 (205:123) that this CNS would be assigned to a gene with a negative loading of Mode 2, corresponding to odds ratio 2.05. Despite its highly significant association with the negative sign of Mode 2 on the hippocampal dataset, the E2FF motif was not significantly associated with the sign of Mode 2 during the cross-system test on the neuronal dataset. The E2FF motif corresponds to a family of 3 matrices describing binding sites for the transcription factors from the E2F family (E2f1–8).

The strong effect of the E2FF motif on expression was specific to Mode 2, as demonstrated by the comparison of the distributions of loadings of modes 1–5 between the E2FF-positive CNSs and the general population of all the CNSs in the hippocampal dataset (supplementary Figure S1 A, see Additional file 1). The same was true when the genes with E2FF were compared to the population of all the genes with at least one CNS (Figure S1 B, see Additional file 1). For the NR2F motif, in addition to the identified effect on the loadings of Mode 2, an effect on the loadings of mode 5 was also observed (supplementary Figure S2, see Additional file 2).

### Pairs of motifs

**Figure 2:**
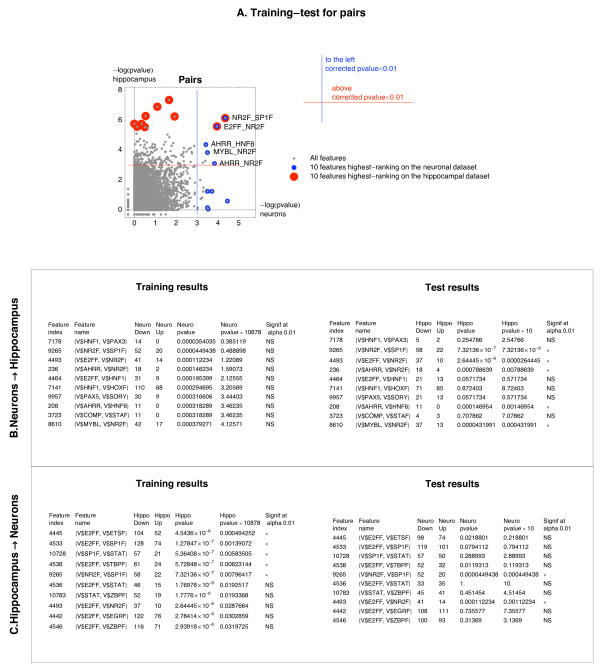
**Identification of pairs of motifs associated with the sign of Mode 2 during hippocampal development and/or neuronal differentiation**. The ten most promising pairs associated with the sign of Mode 2 selected on the neuronal dataset were tested on the hippocampal dataset. The same procedure was repeated for the reverse choice of the training and the test dataset (hippocampal dataset used for training, neuronal for testing). **A**. Illustration of the training-test procedure. For explanation of the procedure – see legend to Fig 1A. **B**. Results of the training on the neuronal dataset and the test on the hippocampal dataset. "Feature name" is a pair of Genomatix "matrix family names" for both motifs. **C**. Results of the training on the hippocampal dataset and test on the neuronal dataset.

When the neuronal dataset was used for training and hippocampal for testing, of the 10 top ranking pairs identified during training, as many as 5 pairs, namely NR2F_SP1F, E2FF_NR2F, AHRR_NR2F, AHRR_HNF6, and MYBL_NR2F scored significant (corrected p-value < 0.01) on the hippocampal dataset (Figure 2A, B).

Notably, the pairs NR2F_SP1F and E2FF_NR2F were identified as the only two pairs significantly associated with down-regulation in the cross-system test for the reverse choice of the training and test dataset (Figure 2A, C). These were the only pairs common between the sets of pairs resulting from the feature selection on the neuronal and on the hippocampal dataset (Figure 2A). The corrected p-values associated with these two pairs were on both datasets smaller than the corrected p-values for the single NR2F motif.

The presence of the NR2F_SP1F pair in a CNS was associated with odds of 2.6:1 (52:20) and 2.64:1 (58:22), on the neuronal and hippocampal dataset, that this CNS would be assigned to a gene with a negative loading of Mode 2. These odds correspond to odds ratios 2.8:1 and 3.24:1, respectively. For the E2FF_NR2F pair the odds for down-regulation were 2.9:1 (41:14) and 3.7:1 (37:10), corresponding to odds ratios 3.16:1 and 4.5:1, on the neuronal and hippocampal dataset, respectively. The SP1F motif corresponds to a family of 6 matrices describing binding sites for 14 transcription factors, including Sp1–8.

Five of the 10 top ranking pairs selected during the training on the hippocampal dataset were significantly associated with the down-regulation on the hippocampal dataset (corrected training p-values < 0.01, Figure 2C). In addition to the two pairs mentioned above that were significant also during the test on the neuronal dataset, the pairs significant on the hippocampal dataset included 3 pairs of E2FF with motifs frequently occurring in the CNSs (SP1F, ETSF, TBPF). The ETSF motif corresponds to a family of 11 matrices describing binding sites for a large number of transcription factors from the Ets family. The TBPF motif corresponds to a family of 5 matrices describing binding sites for the Tata-binding protein factor.

Similarly to the significant motifs, all the significant pairs were associated with down-regulation. The odds for down-regulation observed for the significant pairs were generally higher than for single motifs. The increased odds observed for pairs of motifs that were independently associated with down-regulation (NR2F_E2FF, NR2F_MYBL) could be expected, and were indeed observed, with odds ratios on both datasets of about 3. More interesting was the fact that the presence of the ubiquitous SP1F motif in the NR2F_SP1F pair resulted in an increase of the odds for down-regulation, even though SP1F alone was only weakly associated with down-regulation on the hippocampal dataset, and not at all on the neuronal dataset (Figure 1C). Two pairs, namely AHRR_NR2F, AHRR_HNF6 that were selected on the neuronal dataset and significant in the cross-system test on the hippocampal dataset included AHRR as one motif. The AHRR motif corresponds to a family of 4 matrices describing binding sites for transcription factors Ahr and Arnt. A direct interaction between the transcription factors Ahr/Arnt and Hnf4 on the 3' enhancer of Epo [27] and their joint binding to the promoter of Aldh3 [28] have been described. The motif HNF6 corresponds to a family of 2 matrices describing binding sites for the transcription factors Onecut1–3. These pairs containing AHRR were associated, on both datasets, with high odds for down-regulation, for relatively small sample sizes.

### E2FF versus other motifs

**Figure 3:**
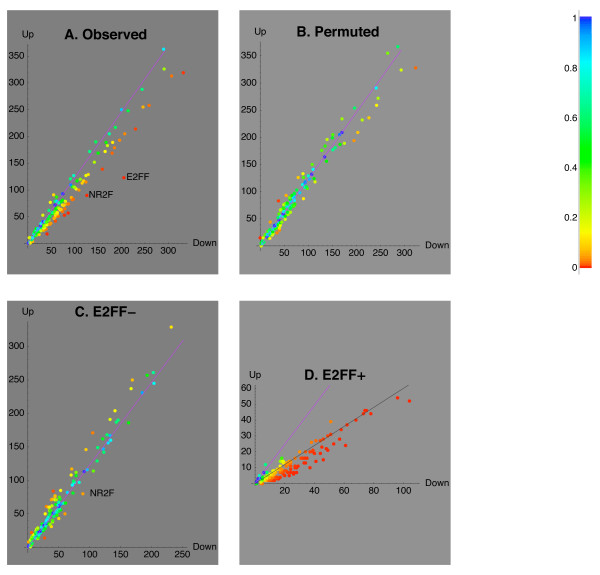
**Dominant effect of the E2FF motif**. Distributions of the signs of loadings of Mode 2 (numbers of CNSs assigned to either sign of loading of Mode 2), in the sets of CNSs selected by the presence of each of the 148 motifs, in the hippocampal dataset. In the panels A-C, a dot represents a set of all the CNSs containing a particular motif, in D – a particular pair of motifs. The X coordinate of the dot is the number of CNSs in this set assigned to the negative sign of Mode 2 (a component of Down-regulation), and the Y coordinate is the number of CNSs in the same set assigned to the positive sign of Mode 2 (a component of Up-regulation). The associated single-test p-value is indicated by the color of each dot. The tangents of the two lines: violet, and black, indicate the ratio of the split expected under the *H*_0 _hypothesis, and the ratio observed for the set of CNSs selected by the presence of the E2FF motif. **A**. Distributions observed for each of 148 motifs, when analyzed in all CNSs. **B**. The same distributions as in A, for a randomized dataset in which the signs of the Mode 2 assigned to each CNS were randomly permuted. **C**. Distributions for each of the 147 motifs other than E2FF, analyzed only among the CNSs without E2FF. **D**. Distributions for each of the 147 motifs other than E2FF, as another motif in addition to E2FF, analyzed only among the CNSs containing the E2FF motif.

The single E2FF motif was most significantly associated with the negative sign of Mode 2 on the hippocampal dataset, and was present in 3 out of 5 significant pairs, with the odds observed for pairs generally higher than for E2FF alone. To explore the interactions between the E2FF motif and other motifs, we analyzed the effect of the presence or absence of the E2FF motif on the distribution of signs of Mode 2 assigned to the sets of CNSs selected by each of the remaining 147 motifs. In Figure 3A, E2FF can be identified as the motif that is most strongly associated with the negative sign of Mode 2 corresponding to down-regulation. However, to our surprise, the majority of the other motifs were also weakly associated with the sign of Mode 2 corresponding to down-regulation. This bias is visible in Figure 3A, as a deviation of the majority of dots to one side of the violet line, indicating (by the tangent of its angle with X axis) the ratio of split expected under *H*_0_. This deviation was data-driven, as confirmed by the same plot for a randomized dataset, in which the expression patterns assigned to the CNSs were randomly permuted (Figure 3B). After excluding several alternative explanations, we found out that the deviation of the majority of motifs from the *H*_0 _disappeared when the CNSs containing the E2FF motif(s) were excluded from the analysis, i.e. when the effect of each of the 147 remaining motifs was analyzed only in the CNSs without E2FF (Figure 3C). Finally, when we analyzed the effect of each of the 147 remaining motifs as another motif in addition to E2FF (only in the CNSs containing E2FF), most motifs enhanced the down-regulatory effect of E2FF, and practically no motif could revert it (Figure 3D). Thus, the weak down-regulatory effect of the majority of motifs (Figure 3A) is explained by the fact that other motifs when co-present with the motif E2FF tend to enhance its down-regulatory effect.

### Application to another dataset

To test the robustness of our novel approach of analysis of *cis*-regulation in a subspace of a single SVD mode of temporal expression profiles, we applied the same methodology to another developmental dataset – the published dataset from the gene profiling of C2C12 myoblast differentiation *in vitro *[29]. This also permitted to test whether the motifs that were associated with down-regulation during neural differentiation would be the same or different to the motifs that are important during the differentiation of cells of the mesenchymal lineage.

#### SVD

**Figure 4:**
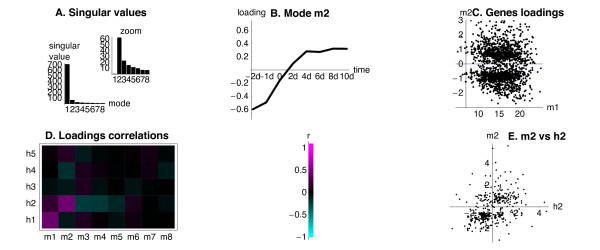
**Results of the SVD on the dataset from gene profiling of myoblast differentiation**. The relevant part of the results of the SVD on the dataset from C2C12 myoblast differentiation *in vitro*, performed as described in Methods. **A**. The singular values for all 8 modes, and for the modes 2–8 to illustrates the importance of the second mode. **B**. Temporal profile (loading onto the time-points) of the second mode. **C**. Loadings of the myoblast modes 1 and 2 (marked m1 and m2) to the expression vectors of individual genes. **D**. Correlations between the loadings of modes resulting from the SVD on the myoblast dataset (marked m1–m8) and the modes resulting from the SVD on the hippocampal dataset (marked h1–h5) calculated for 454 common genes, represented in the colour scale. **E**. Loadings of the respective second modes resulting from the SVD on the myoblast (m2) and on the hippocampal dataset (h2) for the 454 genes common to both datasets.

The treatment of the data and the SVD on the myoblast dataset, were performed as described previously for the hippocampal dataset [30]. The myoblast dataset had 8 time-points, and hence the SVD resulted in 8 modes. Notably, the temporal profiles of the top two most important (Figure 4A) modes were almost identical to the shapes of the top two SVD modes reported before for the hippocampal and the neuronal dataset. The first mode was constant in time (not shown). The second mode represented a component of a monotonous change in expression (followed by a plateau), either up-regulation for the positive loading (Figure 4B), or down-regulation for the negative loading. The distribution of loadings of myoblast modes 1 and 2 was also very similar to the distributions reported before for the hippocampal and the neuronal dataset (Figure 4C). Most importantly, the loadings of the myoblast mode 2 showed a marked correlation (*r *= 0.43) with the loadings of the hippocampal mode 2 (Figure 4D) for the 454 genes common to the two datasets, indicating that many genes changed expression in the same direction during the myoblast differentiation *in vivo *and the hippocampal development *in vitro*. The agreement was higher for the genes with the negative loadings of mode 2 in either system (Figure 4E), many of which are important for cell proliferation (data not shown). This highly significant correlation (t-test p-value of 10^-22^) of the respective modes 2 (thereafter jointly referred to as Mode 2) between the myoblast and hippocampal dataset, suggested that the cross-system approach may also help to identify cis-regulatory features involved in myoblast differentiation, in particular during the exit from the proliferating progenitor cell stage. As before, we used all the genes with CNSs from either dataset during the feature search (see Table 1).

#### Cross-system feature search

The training and testing were performed bi-directionally (myoblast dataset for training and hippocampal for testing, and the other way round), separately for two feature types (motifs and pairs).

**Figure 5:**
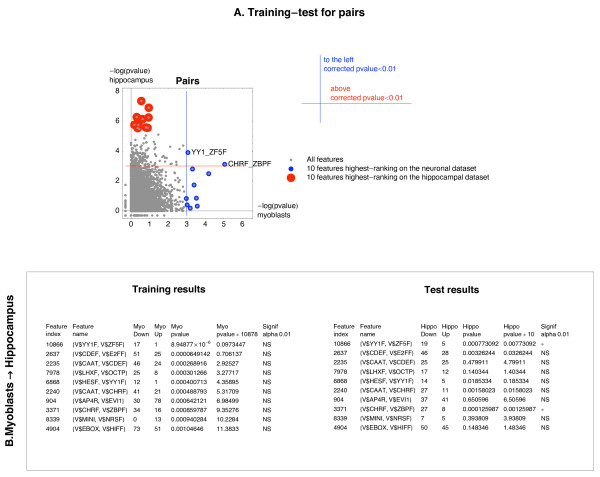
**Identification of pairs of motifs associated with the sign of Mode 2 during myoblast differentiation and hippocampal development**. The ten most promising pairs associated with the sign of Mode 2 selected on the myoblast dataset were tested on the hippocampal dataset. The same procedure was repeated for the reverse choice of the training and the test dataset (hippocampal dataset used for training, myoblast for testing). **A**. Illustration of the training-test procedure. For an explanation of the procedure – see legend to Fig 1A. **B**. Results of a training on the myoblast dataset and a test on the hippocampal dataset, column names as in Figure 1B.

When the myoblast dataset was used for training and hippocampal for testing, 2 pairs, namely YY1_ZF5F and CHRF_ZBPF that were selected during training on the myoblasts dataset scored significant on the hippocampal dataset (Figure 5A). The odds for down-regulation among the CNSs containing the YY1_ZF5F pair were very high on both datasets (17:1, 19:5). The YY1 motif describes binding sites for the transcription factor Yy1. The ZF5F motif describes a binding site for the transcription factor Zfp161. The CHRF motif describes the CDE/CHR tandem element, present in a number of cell cycle-regulated S/G2-specific genes. We note that two more pairs, including CAAT_CHRF would be identified as significant in the cross-system at alpha level 0.05 (Figure 5B). The CAAT motif contains 5 matrices, describing binding sites for the nuclear factor Y (NFY). The CDE/CHR tandem element was shown to interact with the NFY for at least three genes [31].

## Discussion

### Analysis of regulation in a subspace of a single SVD mode

The identification of mammalian regulatory features by the classical "group by expression approach" [32] may be limited by sparse sampling of the relevant expression space. Simultaneously, the number of genes with a particular feature in a dataset may limit the statistical power of the "group by sequence approach" [33] to identify features specific for full temporal expression profiles – because such features are likely to be quite complex (composed of several motifs) and therefore present in relatively few genes.

Here we propose a modification of the "group by sequence approach", which is to search for features associated with expression patterns in a subspace of the original measurements space. Particularly, we report the analysis of *cis-*regulation in a subspace of a single SVD mode of expression, chosen because it was biologically interpretable and conserved between hippocampal development and neuronal differentiation *in vitro*. By employing this approach we identified several features that are associated with down-regulation of gene expression during hippocampal development (here, the most significant was the E2FF motif), or both during hippocampal development and neuronal differentiation (here, the most significant was the NR2F_SP1F pair), or during myoblast differentiation and hippocampal development (here, the most significant was the CHRF_ZBPF pair).

E2FF was identified through the analysis within a single dataset, for the identification of the remaining features crucial was the conservation of Mode 2 between the datasets, which permitted their combined use – resulting in an increased statistical power of the analysis.

The effect size (odds) for most of the features identified by our analysis was moderate, with typical odds for down-regulation of about 3:1. Due to limitations of the microarray technology [4], some profiles may be incorrectly assigned to the genes, with an effect similar to a partial randomization of the dataset. Thus the specificity of the identified features is likely higher than the obtained odds. The odds, and the confirmation rate in the cross-system tests, were generally higher for pairs than for single motifs, in agreement with the postulated role of composite elements [12,13]. For some of the identified pairs a direct interaction between the two motifs (CAAT_CHRF), or between the transcription factors that can bind to them (NR2F_SP1, AHRR_NR2F) has been described. The use of triples of motifs reduced the confirmation rate to 1/10 (data not shown), suggesting that the use of more complex features with the current dataset sizes leads to over-fitting. The confirmation rate was higher for training on the neuronal dataset and testing on the hippocampal dataset than for the reverse choice of the training and the test set.

The identified features account for the sign of Mode 2 of relatively small fractions of all the genes in the analyzed datasets. This was expected, due to the limitations of both the measurement of expression, and of the analysis of *cis*-regulatory regions. Because of a sparse temporal sampling (few time-points in the datasets, leading to the same number of SVD modes) and only partial synchronization of cells, Mode 2 must reflect many mechanisms of regulation, acting through different *cis*-regulatory features, and therefore a single feature cannot account for the loading of this mode for the majority of genes. Our identification of the putative *cis-*regulatory regions was limited only to the CNSs in the upstream -10 kb region, and even in these regions some of the functional motifs were surely missed, and others called incorrectly. Despite all these limitations, our analysis led to identification of features predictive of gene expression during the development of a region of the mouse brain.

The analysis of regulation in subspaces of SVD modes (but without reference to regulatory regions) has been suggested [34] but not reported before. Genes that are likely to be co-regulated by a common regulator and that are functionally related may have different expression profiles due to the action of other regulators. This possibility was noted before, and it was proposed to analyze gene regulation separately for each time-point [7] or experimental condition [35]. Our approach is analogous, but only after the data have been transformed from the original basis (of time-points or conditions) to an orthogonal basis (of SVD modes). Our approach is likely more appropriate if a particular regulator is expected to operate at several time-points.

### Motifs associated with down-regulation during development

We identified the E2FF motif as associated with the negative sign of Mode 2 during hippocampal development, but not during neuronal or myoblast differentiation in cell culture. The transcription factors from the E2F family have a well-established role in the regulation of gene expression during the cell cycle [36]. Neural progenitor cells present in the mouse hippocampus during normal development withdraw from the cell cycle to give rise to neurons or glia [37,38]. Transcription factors from the E2F family are also known to participate in gene silencing of several S-phase genes in fully differentiated neurons [39]. Thus, the regulation of Mode 2 by E2FF may reflect changes in gene expression associated with a progressive withdrawal of neural progenitors from a self-renewing pool and associated with differentiation itself. In the developmental time-window (after the embryonic day 17 – E17) probed by both the neural datasets, the majority of neurons in the hippocampus are already postmitotic, and the cells that still proliferate will become mainly glia. This suggests that the E2FF motif exerted its down-regulatory effect during the hippocampal development after E17 mainly in the glial precursors. This could explain the lack of E2FF association with Mode 2 in the neuronal culture (where Ara-C blocks proliferation of glial precursors).

The finding that many motifs, when co-present in the same CNS, tend to enhance the down-regulatory effect of E2FF suggests that these motifs provide a *cis*-regulatory context, in which the E2FF motif is more likely to have a down-regulatory effect on gene expression. A tendency of functional motifs to cluster in *cis*-regulatory regions is well established [40]. The finding that the down-regulatory effect of E2FF dominated over the effects of any other motif present in the same *cis*-regulatory sequence is interesting, because it seems to be in agreement with a possible role of E2FF in gene silencing during the hippocampal development. The fact that E2FF was not associated with Mode 2 during myoblast differentiation is hard to explain, as a role of E2F in gene silencing of several S-phase genes was demonstrated in this model [41]. A possible hint is offered by the observation of Tomczak et al. [29] that after change to the differentiation medium at day 0, the C2C12 cells transiently up-regulate many cell cycle-related genes. Possibly, the E2F regulation in this system is captured by another mode(s).

We identified the NR2F motif as being associated with down-regulation of gene expression during both the hippocampal development and the neuronal differentiation *in vitro*. This suggests that this motif exerts its role in the differentiating neurons. This possibility is supported by several studies. The NR2F motif is predicted to bind several transcription factors, including the orphan nuclear receptor NR2F1 alias COUP-TF. The transcription factor NR2F1/COUP is a marker of neurogenesis from hydra to vertebrates, postulated to regulate the entry of cells into differentiation [42]. COUP binds to the promoter of nestin in the neural progenitor cells [43]. COUP-TF was also shown to inhibit outgrowth of neurites [44], and is induced upon retinoic acid-induced neural differentiation of the P19 embryonic carcinoma cells [45]. The significance of the NR2F_SP1F pair was higher than of the motif NR2F alone. A possibility of the interaction between the two motifs is corroborated by the fact that the NR2F1 and SP1 proteins were shown to directly interact [46].

All the significant features we identified were associated with down-regulation. When we narrowed the feature selection during the training to select only the features that were associated with up-regulation we were not able to identify any feature that was significant (data not shown). This may reflect the importance of the negative regulation of expression during development [47] and the fact that during development a homogenous population of precursor cells diversifies along many differentiation routes. Consequently, the expression signal associated with the precursor cells is stronger, resulting in an easier identification of features that are associated with expression in the precursor cell status or in the exit from this status. Of the features we identified, E2FF and CHRF may be associated with the cycling progenitor status, while NR2F may be related to the entry into differentiation along the neuronal pathway.

## Conclusion

1. We identified the E2FF motif as a feature significantly associated with down-regulation of gene expression during hippocampal development.

2. We identified the NR2F motif and the NR2F_SP1F pair of motifs as features significantly associated with down-regulation during hippocampal development and neuronal differentiation *in vitro*, suggesting their role in the differentiation of neurons.

3. We demonstrated that the conservation of Mode 2, previously identified between hippocampal development and neuronal differentiation *in vitro*, extends also to the differentiation of myoblasts *in vitro*.

4. Simple features of *cis*-regulatory regions (such as motifs or pairs of motifs) can be predictive of gene expression in a subspace of a single SVD mode.

## Methods

### Sources, annotation and format of expression data

The published datasets from expression profiling of hippocampal development [2], of neuronal differentiation in the mouse hippocampal neuronal culture [4], and of differentiation *in vitro *of the C2C12 myoblasts [29], were mapped to the Ensembl 27_3 gene_stable_ids. We used only the profiles with no missing values from each dataset. This resulted in a mapping of 1926 hippocampal profiles to 1885 gene_stable_ids, of 3216 neuronal profiles to 1824 gene_stable_ids, and of 2895 myoblast profiles to 2008 gene_stable_ids. 453 genes were common between the hippocampal and the neuronal dataset and 454 genes were common between the myoblast dataset and the hippocampal dataset. Separately for either dataset, we computed a single average expression profile for each gene_stable_id, resulting in the following expression matrices: hippocampal *D*_*H *_(1855 genes × 5 time-points), neuronal *D*_*N *_(1824 genes × 6 time-points) and myoblast *D*_*M *_(2008 genes × 8 time-points). The matrices *D*_*H*_, *D*_*N *_and *D*_*M *_were each column-normalized (i.e. each column was divided by its vector norm) and then log-transformed, resulting in the matrices *A*_*H*_, *A*_*N*_, and *A*_*M*_.

### SVD analysis and comparison of loadings between two datasets

The SVD analysis, and the comparison of loadings between two datasets, were performed as described before [25]. Briefly, SVD was performed separately on matrices *A*_*H*_, *A*_*N*_, and *A*_*M *_resulting in matrices *u*_*H *_(1855 × 5), *m*_*H*_, *v*_*H*_; *u*_*N *_(1824 × 6), *m*_*N*_, *v*_*N*_; and *u*_*M *_(2008 × 8), *m*_*M*_, *v*_*M *_respectively. The loadings of the respective second mode for every gene in each dataset are given by the entries in the second columns of the matrices *u*_*H*_, *u*_*N *_and *u*_*M*_. Only the signs of the loadings of the respective second mode were used during the feature search.

For the comparison of loadings between the hippocampal and myoblast dataset, from the matrices *u*_*H *_and *u*_*M *_we selected the gene loadings vectors for the 454 genes common between these two datasets. This resulted in matrices *u*_*HM *_and *u*_*MH*_. Column *k *of matrix *u*_*HM *_contains the loadings of the *k*-th hippocampal mode for all the common genes. Column *l *of matrix *u*_*MH *_contains the loadings of the *l*-th myoblast mode for all the common genes. We calculated the Pearson correlation coefficient *r *between each pair of columns of *u*_*HM *_and *u*_*MH*_.

### Selection of putative regulatory regions

We used conserved non-coding sequences (CNSs) between mouse and human as putative regulatory regions. For each mouse-human orthologous gene pair in Ensembl [48] release 27, the 10 kb orthologous sequences upstream of the annotated transcription start site were aligned using the AVID alignment algorithm [5]. Sequence windows at least 100 bp long with at least 75% identity were selected as candidate regulatory regions. This resulted in the identification of 2021 CNSs for 764 of the 1885 genes in the hippocampal dataset, 2516 CNSs for 897 of the 1824 genes in the neuronal dataset, and 2733 CNSs for 918 of the 2008 genes in the myoblast dataset. The average number of CNSs per gene for the whole Ensemble dataset was 2.7 +/- S.D. = 2.4 CNSs per gene, of average length 185 bp +/- S.D. = 128 bp. All the CNSs identified for each dataset were used for the identification of transcription factor binding motifs.

### Motif identification

Transcription factor binding sites were predicted for all the vertebrate position weight matrices of the Genomatix Matrix Family Library version 6.0 using the program MatInspector [49,50]. This was done in all the CNSs, separately for the mouse and the human sequence of each CNSs pair. Default thresholds, optimized for each motif as described in [26] were used. The motif library contained 431 vertebrate positional weight matrices grouped into 148 matrix families [26].

### Feature definition

Motifs identified with matrices belonging to the same matrix family were treated as the same non-redundant (n-r) motif. The use of n-r motifs allowed a considerable reduction of the number of features, and increased the number of CNSs containing each feature. A CNS was defined to contain a conserved n-r motif X if both the mouse and the human sequence of this CNS pair contained a nonzero number of instances of X (not necessarily in the same AVID-aligned position). A pair, designated X_Y, of conserved n-r motifs X and Y was defined as a simultaneous instance of X and Y in the same CNS. The distance of motifs constituting a pair was limited by the typical length of a CNS (see above). Unless indicated otherwise, in the main text we use the words: "motif" and "pair", in the sense of: "conserved non-redundant motif" and of their pair.

### Binomial model linking features with the sign of Mode 2

In the search for the features associated with the sign of Mode 2, each CNS was assumed to be independent and was assigned the sign of the loading of Mode 2 of its associated gene. In our binomial model, each CNS with an instance of a feature constitutes a binomial trial, in which the positive sign of the loading of Mode 2 for the associated gene is a success, and the negative sign of the loading is a failure. The number of trials for each feature is equal to the number of the CNSs with this feature in a given dataset. The probability *s *of success in a single trial, under the *H*_0 _hypothesis of no association, is equal to the ratio of the number of CNSs assigned to the genes with the positive loadings of Mode 2 to the number of all CNSs in a given dataset (*s*_*H *_= 1115/2021 = 0.55, *s*_*N *_= 1306/2516 = 0.52, and *s*_*M *_= 1532/2733 = 0.56, for the hippocampal, neuronal and the myoblast dataset). From this binomial model we computed two-sided p-values, defined as: the cumulative probability, under *H*_0_, of all the numbers of successes equally or less likely than the observed number (i.e. the cumulative probability of all possible splits as likely or less likely than the observed split). Unless indicated otherwise, in the main text we use the word "p-value" in the sense of: "uncorrected single test p-value".

## Authors' contributions

MD conceived of the study, carried out the analysis, and drafted the manuscript. SA performed the AVID alignments and revised the manuscript. BK interpreted the results and revised the manuscript. All authors read and approved the final manuscript.

## Supplementary Material

Additional File 1 supplementary Figure S1. Effect of the E2FF motif on distributions of loadings of all hippocampal SVD modes. For each mode resulting from the SVD on the hippocampal dataset, we compared its distributions of loadings between the CNSs containing the E2FF motif and the general population of all CNSs in the hippocampal dataset. The same comparison was also performed between the genes containing the E2FF motif (in any CNS) and the general population of all the genes with CNSs in the hippocampal dataset. A. Comparison of the distributions of loadings for the CNSs. B. Comparison of the distributions of loadings for the genes. Click here for file

Additional File 2 supplementary Figure S2. Effect of the NR2F motif on distributions of loadings of all hippocampal SVD modes. The rest of the description as for Figure S1, but with the NR2F motif used to select CNSs and genes. Click here for file

## Figures and Tables

**Table 1:** Partition of genes and putative regulatory sequences between the two signs of Mode 2 loadings. Numbers (#) of genes (gene_stable_ids) and of putative regulatory sequences (CNSs) in the whole datasets, in their common parts, and in their partitions differing by the sign of loadings of Mode 2. The ratios of split used as the general population odds for down-regulation are shown in bold.

Dataset	Number of genes, of which #negative:#positive = ratio of split	Number of genes with CNSs, of which #negative:#positive = ratio of split	Number of CNSs, of which #negative:#positive = ratio of split (odds)
Neuronal dataset (N)	1824 860:964 = 1:1.12	897 422:475 = 1:1.13	2516 1210:1306 = **1:1.08**
Hippocampal dataset (H)	1885 1051:834 = 1.26:1	764 359:405 = 1:1.13	2021 906:1115 = **1:1.23**
Common between N and H	453	223	562
Myoblast dataset (M)	2008 1052:956 = 1.1:1	918 451:467 = 1:1.04	2733 1201:1532 = **1:1.27**
Common between H and M	454	216	610
