# Supplementary figures and images for: Prediction of a key role of motifs binding E2F and NR2F in down-regulation of numerous genes during the development of the mouse hippocampus
Source: BMC Bioinformatics. 2006 Aug 2;7:367. doi: 10.1186/1471-2105-7-367 (PMC1560171; doi:10.1186/1471-2105-7-367)

## A. Sequences

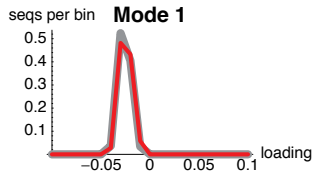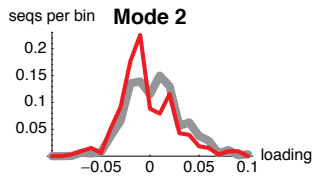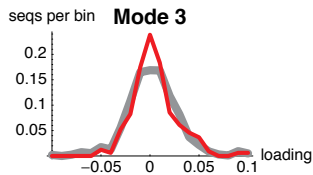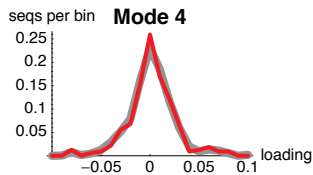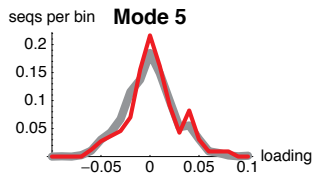

## B. Genes

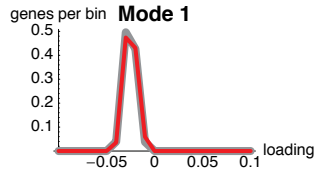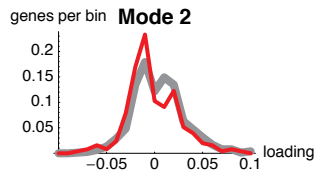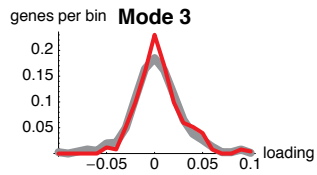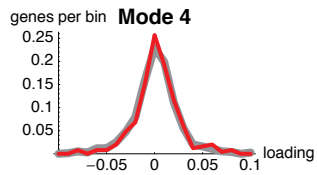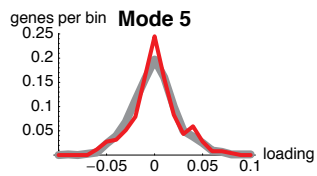

Supplement: Additional File 1 — supplementary Figure S1. Effect of the E2FF motif on distributions of loadings of all hippocampal SVD modes. For each mode resulting from the SVD on the hippocampal dataset, we compared its distributions of loadings between the CNSs containing the E2FF motif and the general population of all CNSs in the hippocampal dataset. The same comparison was also performed between the genes containing the E2FF motif (in any CNS) and the general population of all the genes with CNSs in the hippocampal dataset. A. Comparison of the distributions of loadings for the CNSs. B. Comparison of the distributions of loadings for the genes. [file 1471-2105-7-367-S1.pdf]

## A. Sequences

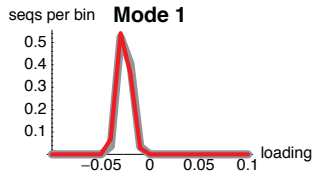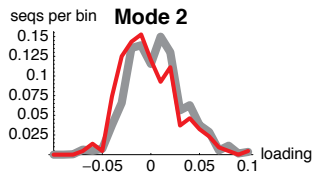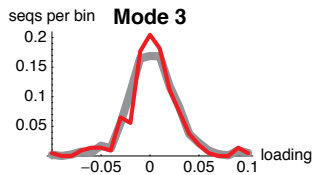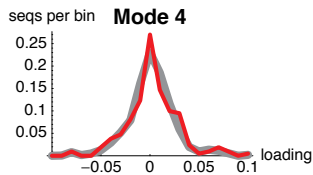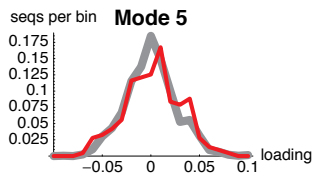

## B. Genes

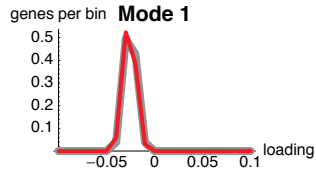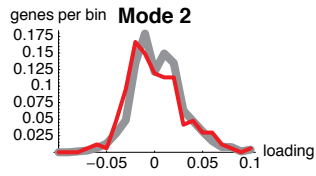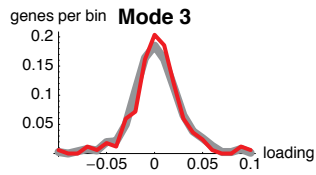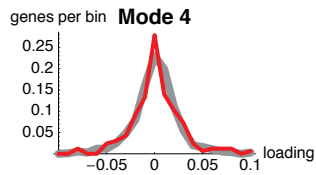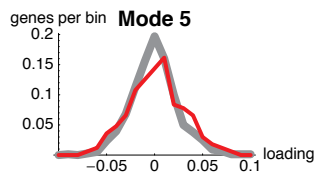

Supplement: Additional File 2 — supplementary Figure S2. Effect of the NR2F motif on distributions of loadings of all hippocampal SVD modes. The rest of the description as for Figure S1, but with the NR2F motif used to select CNSs and genes. [file 1471-2105-7-367-S2.pdf]
